# Jejunal gastric heterotopia presenting as perforation peritonitis in a middle-aged adult: A case report

**DOI:** 10.1016/j.ijscr.2021.02.011

**Published:** 2021-02-09

**Authors:** Wonae Lee, Ye Seob Jee

**Affiliations:** a Department of Pathology, Dankook University College of Medicine, Cheonan, Republic of Korea; b Department of Surgery, Dankook University College of Medicine, Cheonan, Republic of Korea

**Keywords:** Jejunum, Heterotopic gastric mucosa, Ulcer, Perforation, Adult, Case report

## Abstract

**Introduction:**

Gastric heterotopia rarely occurs in the small intestine beyond ligament of Treitz. Most cases of jejunal gastric heterotopia have been reported in children and young adults. Herein we report a case of jejunal gastric heterotopia presenting as a perforation peritonitis in a middle-aged adult.

**Presentation of case:**

A 51-year-old male presented with abrupt onset abdominal pain of 1 day duration. Physical examination revealed abdominal tenderness and rebound tenderness as well as costovertebral angle tenderness. Abdominal computed tomography revealed pneumoperitoneum, suggestive of hollow viscus perforation. At emergency laparotomy, a perforation site was discovered in the jejunum 100 cm distal to the ligament of Treitz. On macroscopic examination, the mucosa contained a 3 × 4 cm ill-defined, shallow ulceration next to the perforation site. Microscopically, the mucosa surrounding the perforation site revealed gastric heterotopia which consisted of gastric foveolar epithelium along with abundant pyloric glands and a few fundic glands.

**Discussion:**

To the best of our knowledge, this case is the presumed oldest jejunal gastric heterotopia patient presenting with perforation peritonitis ever reported.

**Conclusion:**

Jejunal gastric heterotopia should also be considered in the differential diagnosis of perforation peritonitis in adults.

## Highlights

• Jejunal gastric heterotopia rarely occurs and is usually congenital.
• Jejunal gastric heterotopia can be a rare cause of perforation peritonitis in adults.
• Our case is the presumed oldest jejunal gastric heterotopia patient presenting with perforation peritonitis ever reported.

## 1 Introduction

Jejunal gastric heterotopia has been rarely reported. Most cases of jejunal gastric heterotopia tend to be congenital and have been reported in children and young adults [[1], [2], [3], [4], [5], [6]]. Jejunal gastric heterotopia may be asymptomatic or present with intussusception, obstruction, pain, bleeding, ulceration or perforation [[1], [2], [3], [4], [5], [6]]. We report a case of jejunal gastric heterotopia presenting as perforation peritonitis in a middle-aged adult. This case report has been reported in line with the SCARE Criteria [7].

## 2 Presentation of case

**Fig. 1:**
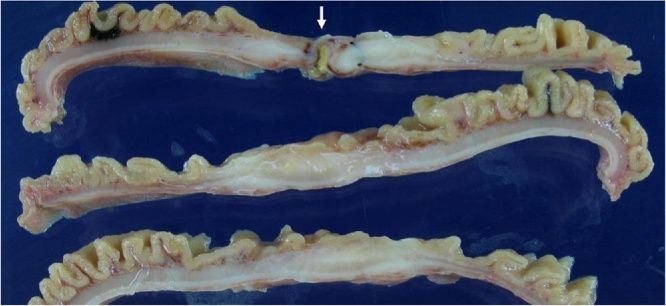
Macroscopically, the sectioned surface of the jejunum shows a perforation site (arrow) with surrounding erosive or ulcerative lesions.

**Fig. 2:**
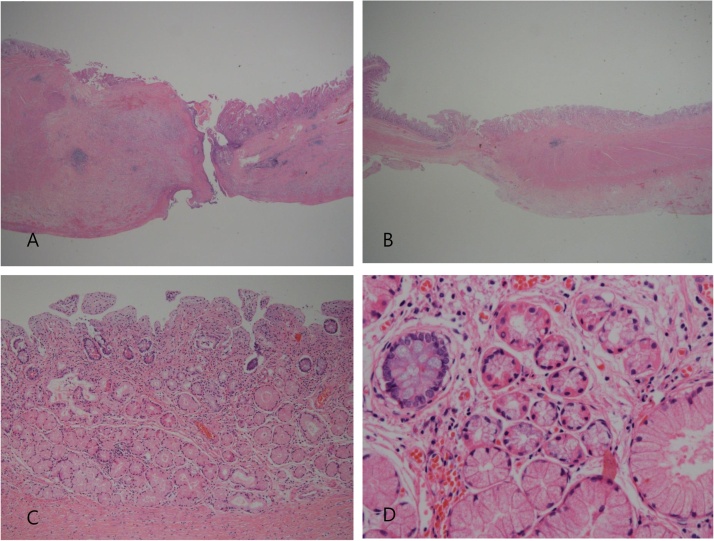
A. Microphotograph of the perforation site shows a perforated chronic ulcer. B. The intestine adjacent to the perforation site reveals heterotopic gastric mucosa with markedly thickened proper muscle and subserosal fibrosis. C. Heterotopic gastric mucosa consists of gastric foveolar epithelium along with abundant pyloric glands and a few fundic glands. D. Higher magnification of heterotopic gastric mucosa highlights fundic glands.

A 51-year-old male presented with abrupt onset abdominal pain of 1 day duration after eating a meal. At that time, the patient has been treated for pulmonary tuberculosis. He has had no drug history, allergies or specific family history. The patient felt some chills and did not have any fever. Physical examination was notable for abdominal tenderness and rebound tenderness as well as costovertebral angle tenderness. Abdominal computed tomography demonstrated pneumoperitoneum suggestive of hollow viscus perforation. Emergency laparotomy was performed by a fully qualified gastrointestinal surgeon in a university hospital. At laparotomy, a perforation site was discovered in the jejunum 100 cm distal to the ligament of Treitz. The size of perforation site measured less than 0.5 cm. Segmental resection of the jejunum with side-to-side anastomosis was performed. The resected jejunal segment measured 8 cm in length which secured appropriate margin of normal mucosa around the perforation site. Macroscopic examination revealed a 3 × 4 cm ill-defined shallow ulceration next to the perforation site (Fig. 1). There was no evidence of an associated diverticulum or intestinal duplication. Microscopically, the perforation site showed a perforated chronic ulcer and peritonitis (Fig. 2A). The intestine around the perforation site revealed heterotopic gastric mucosa with markedly thickened proper muscle and dense subserosal fibrosis (Fig. 2B). The gastric heterotopia composed of gastric foveolar epithelium along with abundant pyloric glands and a few fundic glands (Fig. 2C, D). The heterotopic gastric mucosa was associated with erosions and shallow healed ulcers without any polypoid masses. The pyloric glands were diffusely immunoreactive to the MUC6 antibody. Postoperatively, the patient has had no evidence of gastrointestinal issues or recurrence at 8-year follow-up.

## 3 Discussion

Gastric heterotopia of the gastrointestinal tract occurs commonly in the esophagus and duodenum [8]. Gastric heterotopia of the small intestine beyond the ligament of Treitz is mainly associated with congenital diverticulum and intestinal duplication [9,10]. Gastric heterotopia of the small intestine beyond ligament of Treitz without an associated congenital diverticulum or intestinal duplication is rare [11,12], and most of the cases are reported in children and young adults [[1], [2], [3], [4], [5], [6]]. The most common clinical presentations of jejunal gastric heterotopia are associated with polypoid lesions causing intermittent intussusception [2], gastrointestinal bleeding [3] and obstruction [1]. Peptic ulceration in the area of heterotopia can cause stricture and perforation [13]. Although rare, extensive gastric heterotopia of small intestine can result in massive bleeding, bowel perforation, and death [12].

Gastric heterotopia should be differentiated from gastric metaplasia which is an acquired lesion associated with chronic inflammation and only occupies part of mucosal thickness [6]. Microscopically, gastric heterotopia consists of surface foveolar epithelial cells along with pyloric and fundic glands whereas gastric metaplasia consists of only foveolar epithelial cells without fundic glands [8,11].

Up to our knowledge, the oldest patient with jejunal gastric heterotopia reported in the English literatures was a 52-year-old woman [14] who presented with symptoms of intermittent cramping, abdominal pain, and vomiting due to polypoid mass of jejunal gastric heterotopia . The patient [14] had previously undergone surgery for Meckel diverticulum at age 22. To the best of our knowledge, our case is the presumed oldest jejunal gastric heterotopia patient presenting with perforation peritonitis ever reported. The later clinical presentation in our case may be partially related to the fact that our case presented as a non-mass forming lesion with only tiny amount of parietal cells.

## 4 Conclusion

Although jejunal gastric heterotopia rarely occurs, it should also be considered in the differential diagnosis of perforation peritonitis in adults.

## Declaration of Competing Interest

The authors report no declarations of interest.

## Sources of funding

None.

## Ethical approval

No need for ethical approval.

## Consent

Written informed consent was obtained from the patient’s parents for publication of this case report and accompanying images. A copy of the written consent is available for review by the Editor-in-Chief of this journal on request.

## Author contribution

Wonae Lee: Study concept or design, data collection, writing the paper.

Ye Seob Jee: Primary operator, study concept or design.

## Registration of research studies

Not applicable.

## Guarantor

Wonae Lee.

## Provenance and peer review

Not commissioned, externally peer-reviewed.
